# Differential decay of multiple environmental nucleic acid components

**DOI:** 10.1038/s41598-025-12916-5

**Published:** 2025-07-23

**Authors:** Pedro FP Brandão-Dias, Megan Shaffer, Gledis Guri, Kim M. Parsons, Ryan P. Kelly, Elizabeth Andruszkiewicz Allan

**Affiliations:** 1 School of Marine and Environmental Affairs, University of Washington, Seattle, WA 98105 USA; 2 Conservation Biology Division, Northwest Fisheries Science Center, National Marine Fisheries Service, National Oceanic and Atmospheric Administration, Seattle, WA 98112 USA

**Keywords:** Removal, Bayesian, Mesocosm, Marine mammal, Puget sound, PCR-based techniques, Molecular ecology, Marine biology

## Abstract

Detections of environmental nucleic acids (eNA), such as DNA and RNA, are powerful tools for monitoring biodiversity. Yet, precise interpretation of these indirect detections requires understanding of eNAs persistence. We conducted a decay experiment to track degradation of six eNA components derived from the bottlenose dolphin *Tursiops truncatus*: mitochondrial eDNA of varying lengths, ribosomal eRNA, and messenger eRNA. Target eNAs were quantified over seven days via digital droplet PCR (ddPCR). Decay followed a biphasic exponential model with rapid initial loss (~ 24 h at 15 °C), followed by slower degradation. Mitochondrial messenger eRNA was least stable, disappearing within four hours. Ribosomal eRNA persisted longer but degraded slightly faster than its eDNA counterpart (decay rate λ₁ = 0.236 vs. 0.165 h⁻¹). Longest eDNA fragments decayed more rapidly (λ₁ = 0.190 h^−1^) than shorter ones (λ₁ = 0.114 h^−1^). These findings support using eDNA fragment length as a proxy for degradation and reinforce that combining multiple eNA components with distinct stabilities can provide a molecular clock to infer eNA age. This approach improves the spatiotemporal resolution of eNA-based monitoring, particularly for rare cetaceans that act as point sources. We also emphasize the importance of explicitly distinguishing between RNA types (ribosomal vs. messenger) in environmental studies, given their divergent stability and interpretability.

**Supplementary Information:**

The online version contains supplementary material available at 10.1038/s41598-025-12916-5.

## Introduction

Environmental DNA (eDNA) and, more recently, environmental RNA (eRNA)—collectively referred to as environmental nucleic acids (eNA)—have emerged as powerful tools for ecological monitoring and biodiversity assessments^1,2^. Unlike traditional direct observation methods, eNA-based surveys rely on indirect detection of species through trace amounts of genetic material that organisms release into their environment^3^. Due to the eNA accumulation in space, eNA surveys are fundamentally integrative, containing genetic information accumulated over time rather than providing an instantaneous snapshot. This integrative property enhances detection sensitivity compared to nets or visual surveys which may integrate over space but reflect a single point in time. That is, they are snapshot-based methods, making eNA detection analogous to a short movie rather than a single photographic frame. However, the extent to which eNA surveys integrate species presence over both time and space depends on environmental and molecular factors that govern its persistence and movement, thus making the integration variable^4^.

Accurately interpreting eNA detections requires understanding how much time and space is integrated into each detection event. This, in turn, demands detailed knowledge about the origin, state, transport, and persistence (or decay) of nucleic acids within the environment, collectively referred to as the ecology of eNA^3,5^. Among these processes, the decay rate of eNAs has been among the most frequently estimated parameters^6–8^. The prominence of decay rate studies stems not only from the relative ease of estimating decay rates compared to transport or production, but also from the fundamental role decay plays in determining the temporal resolution of eNA surveys. While transport primarily governs the spatial resolution of detections^9^, largely shaped by hydrological, oceanographic, or atmospheric processes, decay dictates how long genetic signals remain detectable, helping set the temporal bounds of the eNA movie, which reflects a steady-state balance among production, transport, and degradation.

Beyond simply establishing an upper temporal limit for eNA detection, certain applications require more precise aging of the biological source of detections within a sample. This is particularly relevant for point sources or rare detections, such as those of marine mammals^10^ or invasive species^11^. To address this, previous research has proposed using the ratio of eRNA to eDNA as a “molecular clock” for eNA persistence^12^. Since eRNA generally degrades faster than eDNA, qualitatively, a sample with a high proportion of eRNA to eDNA suggests a recent biological source, whereas a sample containing only eDNA indicates an older signal^8,12^. This is analogous to applications in the forensic literature, where the ratio of presence between messenger RNA and DNA, as well as the degradation state of RNA, can be reliably used to infer time since death^13^.

Nonetheless, this framework extends beyond eDNA/eRNA ratios to any eNA component with distinct degradation rates. Environmental nucleic acids exist in multiple molecular forms, including intracellular, particle-adsorbed, dissolved, nuclear, mitochondrial, and other fractions^14–16^. Provided these components decay at different rates, then their relative proportions over time and space provide a molecular signature for estimating detection age^17^. Naturally, this approach can be applied not only to molecular forms of eDNA, but also to experimentally defined fractions such as fragment lengths assessed through multiple genetic markers^18,19^, variations in eNA particle size distributions captured by sequential filtration^20,21^, differences between eNA extracted from distinct environmental media, such as sediment versus water^22^, eDNA to eRNA ratios^12^, among many others.

In theory, the most effective molecular components for estimating detection age are those with strongly contrasting decay rates, as their relative proportions shift measurably over short timescales, thus enhancing spatiotemporal resolution. However, in practice, the choice of eNA components must balance temporal sensitivity with logistical and methodological feasibility. Numerous studies have measured eNA decay rates, consistently highlighting that these rates are highly context dependent. Decay dynamics vary substantially with environmental conditions such as biological activity and temperature^23,24^, depend on target organism^25^, and differ significantly due to methodological differences such as filter pore size and molecular marker length^17,20,26^. Consequently, generalizing decay rates across systems or conditions is challenging, especially since controlled experimental setups seldom fully represent the complexities of natural environments. As a result, accurate molecular time inference requires decay estimates tailored to or closely approximating the specific environmental conditions and molecular targets of interest.

In freshwater systems, multiple studies have compared eRNA and eDNA decay, generally finding that eRNA—particularly messenger RNA (emRNA)—decays more rapidly than eDNA^27,28^. In marine environments, however, relatively few studies have directly compared eRNA and eDNA decay. For example, Qian et al.^29^ observed significantly faster decay of prawn-derived emRNA compared to eDNA, particularly under colder temperatures. Conversely, Wood et al.^30^ examined decay rates of worm- and tunicate-derived emRNA, finding only slightly elevated decay rates compared to eDNA. Similarly, Scriver et al.^31^ found no significant difference in decay between emRNA and eDNA from marine worms. To date, no studies have directly assessed decay rates of ribosomal eRNA (erRNA) in marine environments, although Miyata et al.^32^ noted that erRNA yielded more ecologically relevant metabarcoding detections than eDNA, suggesting greater transience and thus potentially faster decay. Crucially, despite this emerging body of work on eRNA, no studies have evaluated the decay rates of eNA components, eDNA or eRNA, specifically derived from marine mammals.

**Fig. 1:**
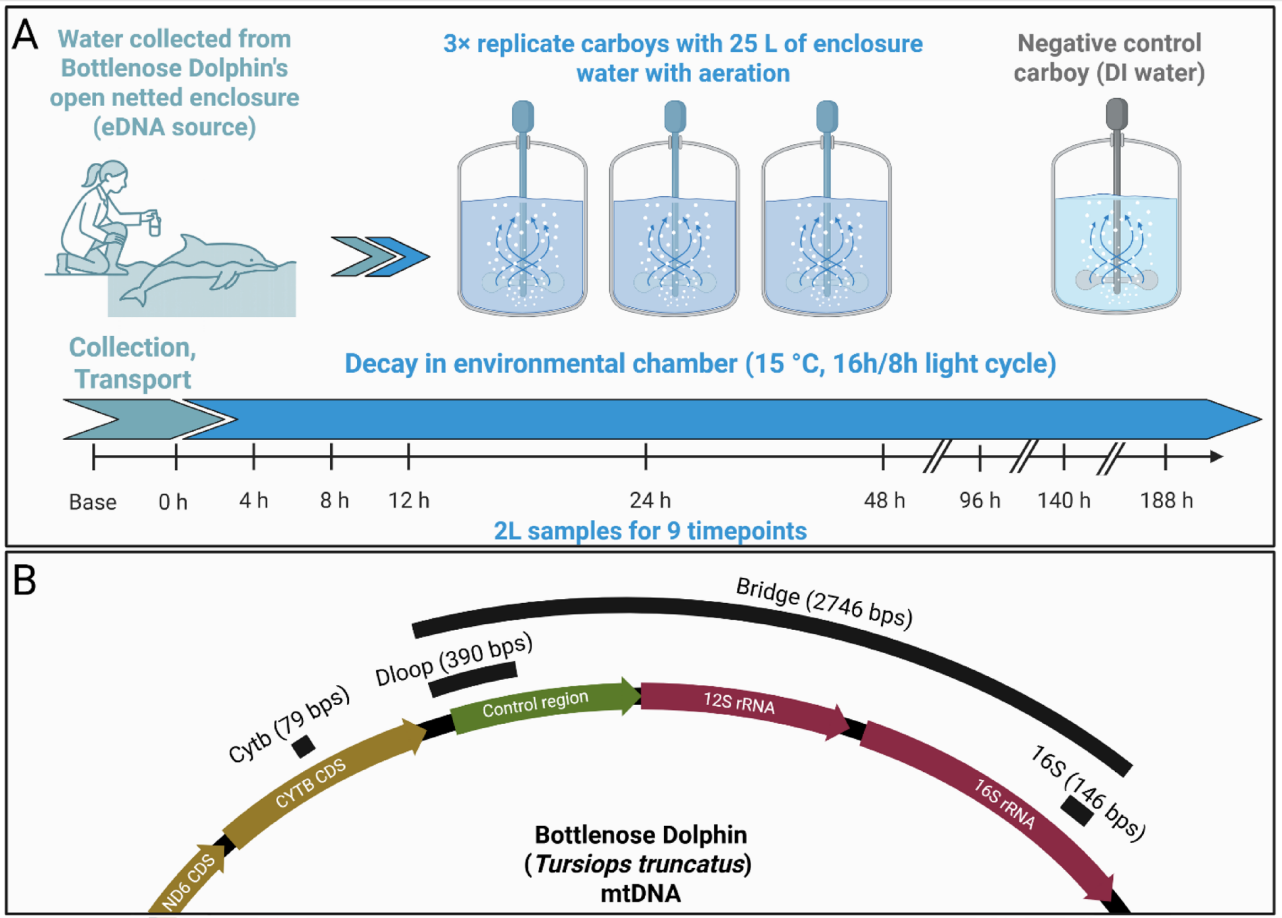
Overview of the experimental design and the four mitochondrial targets quantified. (**a**) Water-decay experiment. Seawater was scooped from inside an open-net enclosure housing an atlantic bottlenose dolphin (*Tursiops truncatus*) and poured into three 25 L carboys (biological replicates). A fourth carboy containing de-ionized (DI) water served as cross-contamination control. Carboys were held in a 15 °C environmental chamber with continuous aeration and a 16 h light/8 h dark cycle. Two-liter subsamples were withdrawn at nine timepoints (0–188 h) for size-fractionated filtration and downstream DNA/RNA extraction. (**b**) Locations of the markers within the bottlenose dolphin mitochondrial genome. Black boxes show the relative positions of the 79 bp Cytb amplicon (protein-coding), the 390 bp D-loop amplicon (non-coding control region), and the 146 bp 16 S rRNA amplicon (ribosomal). The 2746 bp segment spanning D-loop to 16 S (black arc labelled “Bridge”) represents the minimum relatively intact bridging fragment between 16 S and Dloop loci, which we have quantified using duplex ddPCR, Fig S1. Figure created in https://BioRender.com.

Here, we experimentally assessed the differential decay rates of multiple eNA components from the common bottlenose dolphin, *Tursiops truncatus* (Montagu, 1821). Using seawater sourced from an open environment, netted dolphin enclosure, we conducted a controlled decay experiment, tracking the persistence of eDNA of several molecular lengths, emRNA, and erRNA over time (Fig. 1). We hypothesized that eRNA would degrade faster than eDNA, with emRNA decaying the fastest due to its transient nature. We expected erRNA to persist longer than emRNA due to its structural properties, but still degrade more rapidly than eDNA. Lastly, we anticipated that larger DNA fragments would decay faster than shorter ones, following previous findings^20,33^. By quantifying these decay rates, we aimed to improve the temporal resolution of marine mammal detections and refine our understanding of eNA persistence in marine environments.

## Results

### Controls

All target eNA assays from the fourth (control) carboy, filtration negative controls, extraction blanks, PCR no template controls, and no reverse transcriptase (No-RT) controls yielded zero positive droplets, with the exception of occasional single positive droplets in No-RT controls. These rare events indicate minimal residual DNA carryover in RNA extracts despite DNase treatment. To account for this, we subtracted the droplet count observed in each No-RT control from the corresponding eRNA droplet count of the same sample before proceeding with downstream analyses.

### Little eDNA found in smaller pore sizes

**Fig. 2:**
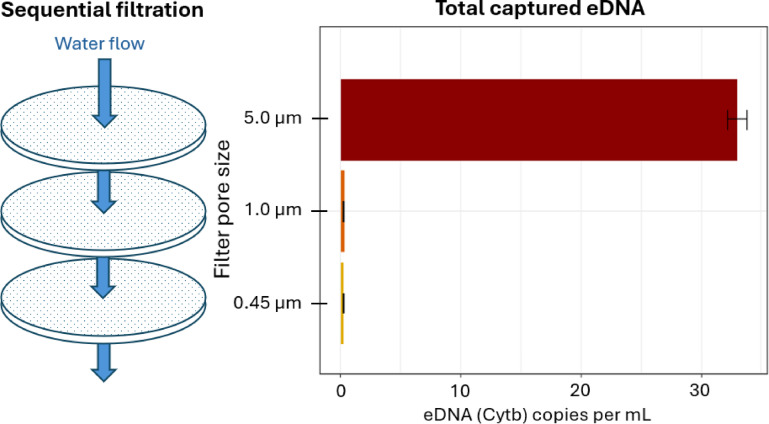
Particle size distribution of Bottlenose Dolphin eDNA in the first timepoint. Concentration of dolphin mitochondrial Cytb eDNA recovered following sequential filtration at the first sampling time-point as a function of pore size. Filters with pore sizes < 5 μm retained negligible eDNA, so subsequent analyses rely exclusively on extracts from the 5 μm fraction.

To assess the particle-size distribution of dolphin eDNA, we filtered each time-point sample through a 3-stage serial filtration system (5 μm, 1.0 μm, 0.45 μm), representing filter sizes commonly used in eNA literature^9,34^. Preliminary sample screening of the first timepoint samples found that > 95% of the Cytb signal was captured on the 5 μm filter at time zero. Therefore, all downstream analyses used this fraction exclusively (Fig. 2).

### Cytb messenger RNA was short-lived, and eNA decay was biphasic

We quantified decay of six different eNA components, including four eDNA markers of varying length (Fig. 1b), one erRNA marker, and one emRNA marker (Table 1).

**Table 1:** Markers analyzed herein. “Bridge” refers to the 16 S-Dloop Bridge sequence (Figs. 1b, S1).

Mitochondrial locus	Nucleotide type	Extract	Assay	Target length (base pairs)
Cytb	DNA	eDNA	Cytb monoplex	79
Cytb	mRNA	eRNA	Cytb monoplex	79
16 S	DNA	eDNA	16 S-Dloop duplex	146
16 S	rRNA	eRNA	16 S monoplex	146
Dloop	DNA	eDNA	16 S-Dloop duplex	390
Bridge	DNA	eDNA	16 S-Dloop duplex*	2746–16,390

* See supplement for calculation details.

**Fig. 3:**
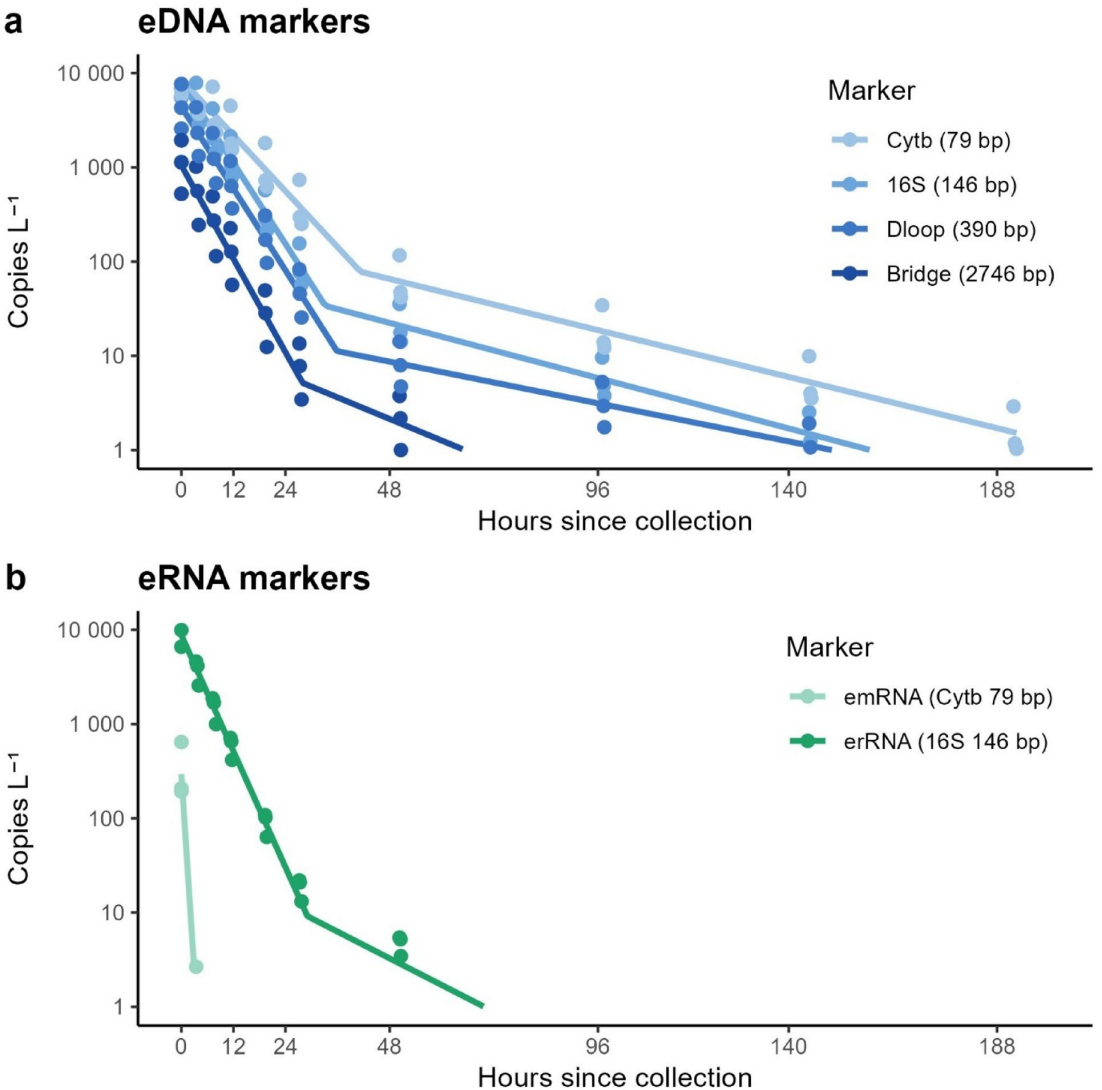
Biphasic-model decay of dolphin environmental nucleic acids (eNAs). (**a**) Mitochondrial DNA targets. (**b**) RNA targets. Solid lines are the posterior mean trajectories from the biphasic Bayesian ddPCR model; filled circles are the model-predicted concentrations at each sampling time for every biological replicate (three carboys). Colors distinguish markers and correspond to amplicon length: Cytb 79 bp, 16S 146 bp, DLL1 390 bp, and the 2 746 bp Bridge (DNA); Cytb emRNA 79 bp and 16S erRNA 146 bp (RNA). Axes are log-scaled; concentrations are expressed as copies L⁻¹.

We detected dolphin-derived eDNA and 16 S ribosomal eRNA across multiple timepoints, with signals persisting up to 48 h after the start of the experiment. In contrast, Cytochrome b (Cytb) messenger eRNA was only detected in the first pre-transport sample (Figs. 3, S4). This signal was lost during the ~ 3-hour transport period, and no Cytb emRNA was detected in subsequent laboratory samples, suggesting extremely rapid degradation. For eDNA, molecular length was a strong predictor of persistence (Fig. 3a). Shorter targets, such as Cytb (λ₁ = 0.114 h⁻¹), decayed more slowly, while longer targets like the Bridge fragment spanning 16 S and D-loop decayed faster (λ₁ = 0.190 h⁻¹).

Therefore, decay rate estimates varied by marker (Table 2). Although Cytb emRNA showed the fastest apparent initial decay (λ₁ = 1.615 h⁻¹), this estimate is based on a single detection and subsequent non-detections, and should be interpreted with caution. Among consistently detected markers, 16 S erRNA had the highest initial decay rate (λ₁ = 0.236 h⁻¹; Fig. 3b).

**Table 2:** Posterior decay rates estimated across markers. “λ_1_” is the decay rate for the first phase of the exponential decay; “λ_1_ 2.5%” and “λ_1_ 97.5%” represent it’s 95% confidence interval; “t_x_” is the time break at which the decay rate was found to change from the first phase to the second; and “λ_2_” is the decay rate for the second phase of the exponential decay. NA values indicate no detections were made at the relevant timepoint, making values unidentifiable.

Marker	λ_1_	λ_1_ 2.5%	λ_1_ 97.5%	t_x_	λ_2_
Cytb eDNA	0.114 h^−1^	0.110 h^−1^	0.118 h^−1^	41 h	0.026 h^−1^
Cytb emRNA	1.615 h^−1^	0.877 h^−1^	2.763 h^−1^	NA	NA
16 S eDNA	0.165 h^−1^	0.161 h^−1^	0.169 h^−1^	33 h	0.028 h^−1^
16 S erRNA	0.236 h^−1^	0.207 h^−1^	0.267 h^−1^	29 h	0.054 h^−1^
Dloop eDNA	0.166 h^−1^	0.160 h^−1^	0.173 h^−1^	36 h	0.021 h^−1^
Bridge eDNA	0.190 h^−1^	0.175 h^−1^	0.206 h^−1^	28 h	0.044 h^−1^

Beyond this specific marker behavior, we observed that environmental nucleic acids (eNA) generally followed a biphasic decay pattern. This was especially clear for short eDNA fragments, which showed a steep decline within the first 48 h, followed by a plateau phase in which low concentrations persisted through the end of the 7-day experiment. To formalize this observation, we compared six candidate decay models (Supplement), and the biphasic exponential model provided the best fit to the data based on leave-one-out cross-validation (Table S3). The modelled transition time between the two decay phases consistently fell between 24 and 48 h, although the exact value was prior dependent, as no samples were taken between these times. All targets exhibited a slower decay rate on the second phase (λ_2_ between 0.054 and 0.021, Table 2).

### Molecular clock behavior in fast decaying eNA components

**Fig. 4:**
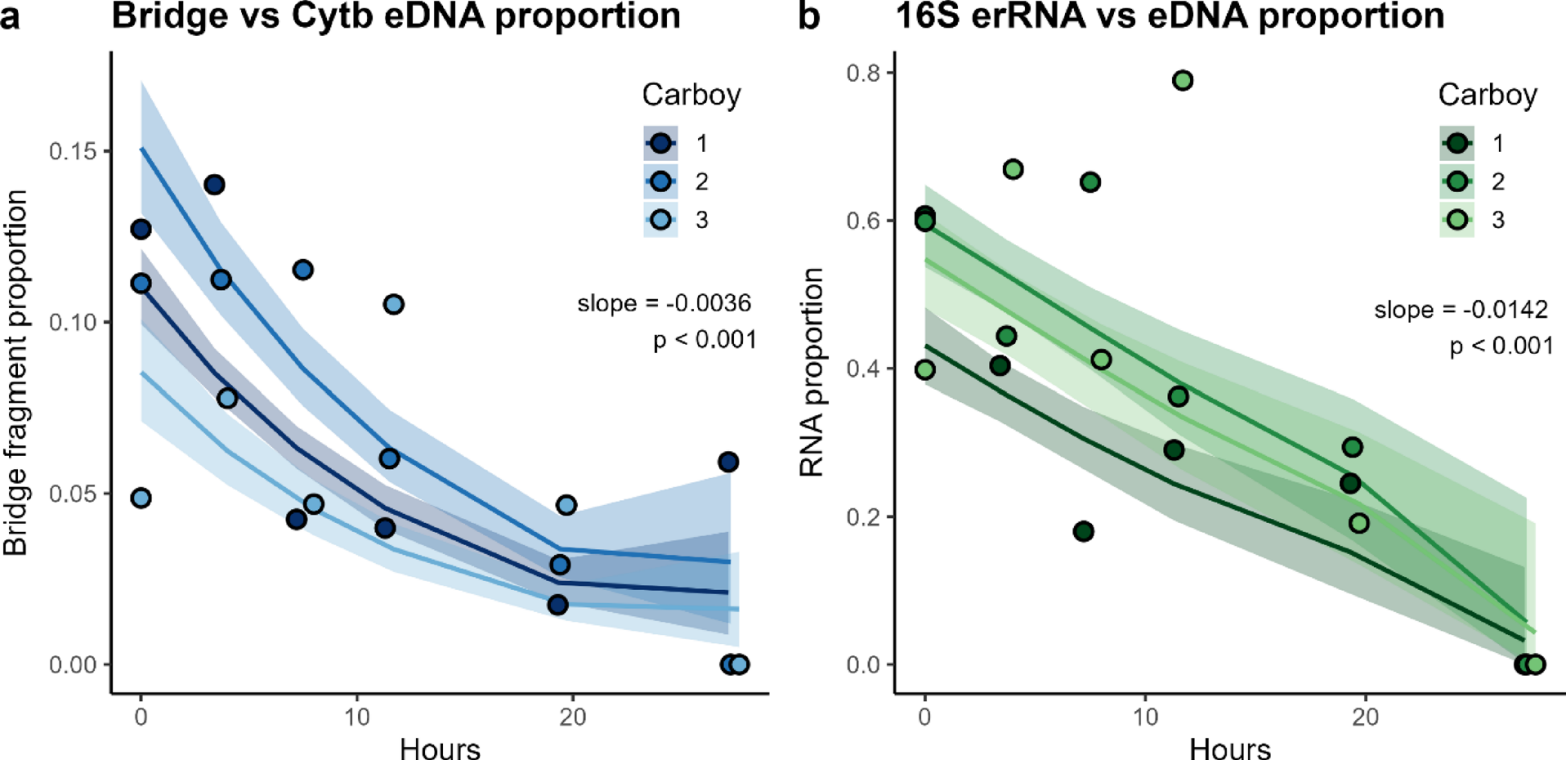
Time-sensitive molecular proportions based on posterior predictions from the biphasic decay model. (**a**) Proportion of long (Bridge) mtDNA fragments relative to total Cytb fragments over time; (**b**) proportion of 16 S ribosomal erRNA relative to total 16 S nucleic acids (eDNA + erRNA) over time. Solid lines represent posterior median trajectories for each carboy. Shaded ribbons are 95% credible intervals (2.5th–97.5th percentiles). Hollow circles show individual ddPCR replicate measurements at each time point, colored by carboy. Linear regression slopes and p-values, fit to the pooled posterior median trajectories (across all carboys), are annotated in each panel.

The proportion of long (Bridge) fragments declined significantly over the first 24 h (slope = − 0.0036 h⁻¹, *p* < 0.001; Fig. 4a), consistent with faster degradation of longer molecules. Similarly, the ribosomal erRNA∶eDNA ratio (16 S marker) also decreased over time (slope = − 0.0142 h⁻¹, *p* < 0.001; Fig. 4b), reflecting the faster degradation of rRNA relative to DNA. These consistent trends suggest that both ratios may serve as coarse indicators of molecular age in environmental samples. However, the high variability in the observed ratios limits temporal resolution to broad scales, probably on the order of tens of hours.

## Discussion

This study provides the first empirical estimates of decay rates for multiple environmental nucleic acid (eNA) components derived from a marine mammal. Using a controlled experiment with seawater from a dolphin enclosure, we documented the degradation of mitochondrial eDNA fragments of different lengths, ribosomal eRNA, and messenger eRNA from *Tursiops truncatus* over seven days. We found that decay patterns were highly dependent on molecule type and length, with messenger RNA degrading the fastest, ribosomal RNA decaying slightly faster than its DNA counterpart, and longer DNA fragments decaying more rapidly than shorter ones. Across most targets, decay followed a biphasic trajectory, with a steep initial decline and a slower second phase. These results confirm that eNA components differ markedly in environmental stability and underscore the potential of combining multiple markers to improve the temporal resolution of eNA-based detections.

### Rethinking molecular strategies for eDNA age Estimation

The ratio between eDNA and emRNA has been proposed as a molecular proxy for time since eDNA shedding^12^. That is, assuming a single point source or shedding event, the amount of emRNA relative to DNA can be used as a “molecular clock” to determine spatiotemporal distance between sample and source, such that high RNA:DNA ratios indicate recent activity. This is consistent with the spatiotemporally variable eDNA composition hypothesis^17^, which states that as eNA travels away from its source, its composition changes predictably, allowing for age estimation. However, our results suggest that emRNA is not well-suited for this application in marine systems given its inconsistent detectability. Cytb emRNA was only detectable at the initial timepoint and degraded beyond detection during transport—highlighting the molecule’s extreme lability. Similar challenges have been noted in other marine studies, where emRNA is often either undetectable or not measurably different in decay from eDNA^30,35^. Beyond this, emRNA analysis requires transcript-specific assays, rapid stabilization, and higher-cost laboratory workflows^2,36^, which limit its practicality for most applications.

Instead, our findings support the use of eDNA fragment length as a proxy for degradation state, which has also been proposed by previous studies^37,38^. Longer mitochondrial markers decayed consistently faster than shorter ones, as shown here and in prior work^20,33^, making them well-suited for relative age estimation as in Fig. 4a^19^. This method is logistically simple, relying on standard eDNA workflows and multiplexed assays, and broadly applicable across taxa and systems. Importantly, quantitative estimation of eDNA age requires both accurate decay rates and knowledge of the initial proportion of each component, so that their relative abundance over time can be meaningfully interpreted. When targeting mitochondrial loci, the physical linkage of gene regions provides a useful simplification: short and long fragments are expected to originate at roughly equal copy number, enabling decay-based inferences without requiring independent normalization of starting ratios. On the other hand, because mRNA expression levels change by context, the starting proportion between mRNA and DNA in environmental samples will always vary, and initial proportions cannot be accurately established. Thus, pairing mitochondrial markers of different lengths potentially offers a scalable, cost-effective alternative to RNA-based strategies. Rather than relying on a single target, a duplex assay can be designed to simultaneously amplify two mitochondrial regions with a deliberate difference in length, maximizing the contrast in decay rates while maintaining amplification efficiency.

Nonetheless, despite the tightly controlled conditions of our experiment, this approach showed several limitations. First, the high variance in the observed proportions of rapidly decaying components means that meaningful temporal resolution is probably only achievable on the scale of tens of hours. Second, the model assumes a single source or point release, which is rarely the case in natural systems. In reality, eDNA samples are expected to contain molecules originating from multiple shedding events over time. Because of this, and due to the exponential nature of decay, molecular age estimates may be biased toward the most recent shedding event or, in some cases, become effectively indeterminable. Third, environmental factors such as temperature and microbial activity modulate decay dynamics in natural settings^24,39^, but these were beyond the scope of this study. Broader generalizations about the applicability of molecular clock approaches for eNA will require multi-factorial studies across diverse systems and taxa.

### Mechanisms and practical significance of biphasic decay

Dolphin eDNA in our experiment followed a biphasic exponential decay: a steep first-phase loss lasting ~ 48 h, followed by a low-level “tail” that persisted for days. Similar two-phase kinetics have been reported from rivers, lakes, and coastal waters^25,40–42^, but often go unrecognized when decay experiments end after only one or two days^e.g. 43^. We detected no compelling biphasic signal for either ribosomal or messenger eRNA, but it is possible RNA also follows the same pattern.

The processes that generate two-phase decay remain unresolved. Three non-exclusive explanations have been previously proposed: (i) physical shielding, in which DNA adsorbed to mineral or organic particles become inaccessible to nucleases^44,45^; (ii) encapsulation, whereby intact cells and mitochondria only release DNA after membrane rupture, thus persisting for longer times^46,47^; and (iii) component-dependent removal, whereby different eNA components decay at different rates, and observed curves are the combination of multiple decay curves. For instance, large aggregates may settle or be grazed quickly resulting in rapid initial decay, while finer particles or dissolved DNA persist in suspension, creating the lagging tail^47^. Given each of these mechanisms are associated with different eDNA states, disentangling them will require experiments that characterize or separate DNA states.

From a monitoring perspective, the slow second phase probably contributes little to routine eDNA surveys: most field detections will be dominated by the highly concentrated but rapidly decaying fraction, as the residual tail is easily swamped by fresh inputs. Nonetheless, understanding the dynamics of this second phase may improve inference about source age, particularly in low-concentration contexts where legacy signals become more detectable. In this study, we focused on the first 24 h of decay, corresponding to the steep initial phase where most molecular loss occurs. Incorporating the second phase into time-sensitive inference is more challenging due to its persistence and potential confounding with recent inputs. However, this limitation can be mitigated by targeting molecular features that either degrade rapidly (e.g., ribosomal RNA) or exhibit limited persistence in the second phase (e.g., very long DNA fragments), both of which may serve as more reliable indicators of molecular age.

### Distinguishing ribosomal and messenger eRNA in environmental studies

In our study, we found cytochrome b (Cytb) messenger eRNA (emRNA) at very low concentrations, detectable only at the initial time point before water samples were transferred to the environmental chamber. Low levels of Cytb emRNA in environmental samples has been found in other studies^48^, and these low concentrations are the likely explained by low generation and expression of Cytb from eNA-generating tissue. In contrast, ribosomal eRNA was present at much higher concentrations in our samples, consistent with its constitutive expression, and decayed only slightly faster than its equivalent DNA. This contrast in stability and concentrations of both forms of RNA aligns with well-documented differences in RNA stability from forensic and molecular biology research, where ribosomal RNA’s secondary structure may protect it from degradation^49,50^. These findings highlight a fundamental distinction between erRNA and emRNA: while erRNA is found in high concentrations and can be reliably detected, emRNA is highly transient, making it a good candidate for detection of more recent targets, but potentially unreliable in yielding consistent taxa detections given its low starting concentrations.

The rapid degradation of mRNA is associated with its biological function. Within cells, mRNA is a highly labile molecule that allows for rapid and dynamic gene expression regulation^51^. To maintain precise control over protein synthesis, cells possess multiple pathways for degrading mRNA efficiently, including exonuclease-mediated decay and other complex biomolecular pathways^52,53^. Additionally, mRNA’s single-stranded structure and chemical composition (i.e., an extra hydroxyl group) make it inherently less stable than DNA, leading to its rapid degradation both within cells and in the environment, particularly in high temperatures and alkaline conditions^27,54^. In contrast, rRNA is constitutively (permanently) expressed in large quantities, and plays a structural role in ribosome assembly in its RNA form, being thus long-lived in the cell. Accordingly, ribosomal RNA often comprises > 80% of the total RNA in the cell—mostly from cytoplasmic ribosomes^55,56^. Within mitochondria, rRNAs also greatly dominate the transcript pool, with rRNA quantities 10–100× higher than mitochondrial mRNAs^57,58^. Its highly structured secondary and tertiary conformations enhance its resistance to enzymatic degradation^50,59^, which likely extends to higher stability in the environment as well.

Despite these well-established molecular differences, the term “eRNA” is often used interchangeably in the eDNA literature to refer to both erRNA and emRNA, despite their fundamentally different properties. This conflation can lead to incorrect assumptions about eRNA persistence, decay rates, and ecological interpretability. Specifically, our results and previous literature indicate that emRNA degrades much faster than eDNA in environmental samples, and its initial concentration depends heavily on gene expression in the tissues shedding eDNA. Because gene expression varies across tissues, developmental stages, and environmental conditions, interpreting emRNA abundance requires transcriptomic knowledge of the target species—information that is often unavailable, but can be used to inform emRNA assay development^60^. Consequently, assays designed for both eDNA and emRNA detection (e.g., targeting Cytb, as tested here) may not be widely effective, as the emRNA marker will have to be customized for each application.

In contrast, erRNA is more abundant, making it a more stable biomarker but unsuitable for most other applications where emRNA would be advantageous over eDNA, such as metabolic inferences^2^. Supporting this, previous metabarcoding studies using erRNA and eDNA to detect species with a 12 S marker found minimal differences in detection rates, though erRNA showed faster species accumulation curves^35,61^. Macher et al.^35^ also tested emRNA-based detection using cytochrome oxidase I (COI), and as expected, emRNA was less effective than eDNA in species detection due to its rapid degradation (see above). However, its faster decay resulted in stronger spatiotemporal patterns, reflecting its short-lived nature in the environment.

These findings reinforce the importance of distinguishing rRNA from mRNA in environmental studies. The ecological patterns observed for rRNA cannot be assumed to apply to mRNA, and vice versa. Given their fundamental biological and functional differences, treating both molecules collectively as “eRNA” is misleading and can obscure key differences in their degradation rates and interpretability in environmental monitoring. Future research should clarify this distinction and ensure that eRNA studies account for the vastly different behaviors of these two RNA types in environmental contexts.

### Marine mammal eNA composition and optimizing detections in the field

Interestingly, we found that when sequentially filtering, the vast majority of dolphin eDNA was captured on the largest pore size filter (5 μm), with only residual amounts recovered in smaller size fractions (Fig. 2). This pattern contrasts with previous studies on the particle size distribution (PSD) of eDNA from other marine taxa, such as teleost fish^21^ and elasmobranchs^62^, whose eDNA was found to be more evenly distributed down to small filter pore sizes (< 1 μm). While eDNA PSD is known to vary with environmental conditions and organism^44,63^, the pattern observed here closely resembles that reported for freshwater fish in the presence of clay or titanium dioxide, which strongly adsorb to eDNA^64^. However, such particles were not present in our samples in substantial quantities.

Three alternative explanations could account for the observed PSD pattern. First, the filtration system may have failed at smaller pore sizes, leading to underrepresentation of eDNA in those fractions. However, this seems unlikely given that the same pattern emerged across three independently filtered replicate carboys. Second, the behavior and provisions of captive dolphins may have influenced their eDNA PSD, making it unrepresentative of wild populations. A similar experiment using wild animals is therefore necessary to validate these findings. However, if experimental error and captivity effects can be ruled out, the results suggest that marine mammal eNA is primarily associated with larger particles—potentially due to tissue origin, mucus binding, or aggregation. If independently confirmed, this has important implications for sampling design: using larger pore size filters may increase eDNA yield and enhance detection probability for marine mammals by enabling the filtration of greater water volumes.

In addition to using larger filter pore sizes, marker choice may also influence detection outcomes in the field. Shorter amplicons, which persist longer and occur at higher concentrations, are better suited for reliable detection under typical degradation conditions. Conversely, longer markers, which degrade more rapidly, may be used strategically to emphasize more recent shedding events, allowing for potential inference of eNA age, or to provide additional information like haplotype and individual identification.

### Carryover DNA and components of eDNA

Commercially available extractions columns were originally designed to extract high molecular yield DNA from tissue samples. However, they have been commonly used for extracting eDNA from filters over the past two decades and have yielded sufficient DNA for most applications^1^. To our knowledge, no other studies have captured and quantified eDNA that passes through the DNA extraction columns to quantify this inefficiency. Here, we found a substantial amount of eDNA passed through the initial column, largely comprising shorter DNA molecules as measured by a TapeStation (Supplement Fig. 2). This finding points to a potential blind spot in many eDNA workflows: studies seeking rare targets or high detection sensitivity should be cautious about relying on column-based extraction methods, despite their convenience and scalability. Nonetheless, further study is needed to verify if this is a systematic issue with column extractions in general or just with the kit used herein.

In addition to quantifying the amount of carryover eDNA, we also quantified the decay rate of carryover eDNA with one marker (Cytb) and found it to decay significantly slower than the eDNA fraction captured on the first extraction column (Fig. S3). This is likely due to a cascading effect, where the decay of longer eDNA molecules creates shorter eDNA molecules, resulting in a slower apparent decay of the smaller fragment size^17^. Nonetheless, it is unclear if this method of splitting eNA components (i.e., eDNA vs. carryover eDNA) will be useful in the future because the fraction of eNA passing through the first column may not be stable, and may vary substantially under varying total quantities of DNA in the sample and other chemical characteristics of the sample. Thus, while splitting eNA by molecule length (in basepairs) may find its applications, separation of multiple eNA components may be more reliable with other methods such as sequential filtration^20,65^, evaluating multiple markers of varying lengths as here, or differential extraction methods^14^.

## Methods

### Environmental NA source and decay experiment

A small, managed group of non-native Atlantic bottlenose dolphins (*Tursiops truncatus*) is housed in a defined area along the eastern bank of Hood Canal, Washington, as part of the U.S. Navy Marine Mammal Program. These dolphins frequently inhabit netted enclosures adjacent to a pier, where seawater freely exchanges with the surrounding marine environment. In October 2024 we collected water from within one of these netted enclosures containing a single dolphin at the time of sampling to use as a source of eNA for the decay study. No live animal procedures were performed in this study. Environmental NA was collected non-invasively from the water within netted enclosure containing a single dolphin, but without any manipulation or disturbance to the animal. All methods were carried out in accordance with relevant guidelines and regulations.

Using a 2-liter pitcher, we filled three replicate 25-liter carboys, which served as the three biological replicates for the decay study (Fig. 1a). To minimize contamination, collectors wore gloves during sampling, and both the pitcher and carboys had been cleaned overnight with 1% bleach and extensively rinsed with deionized water before use. After collection, water within each carboy was mixed, and a 2-liter baseline sample was filtered from each carboy to assess initial eNA concentrations before transport. These samples were immediately filtered and preserved (see below for filtration and preservation details). The carboys were then sealed and transported at ambient temperature to the laboratory and an environmental chamber for the decay experiment. Temperature loggers placed inside one of the carboys recorded transport temperatures ranging from 16 °C to 12 °C over the course of ~ 3 h of transport back to the laboratory.

At the laboratory, the three biological replicate carboys were decontaminated externally with bleach before being placed inside an environmental chamber, where they remained for the duration of the experiment. A fourth carboy, filled with deionized (DI) water, was also placed in the environmental chamber as a cross-contamination control. The environmental chamber was maintained at a constant 15 °C with a 16-hour daylight cycle throughout the experiment. To ensure water circulation, we inserted air hoses with stones powered by a Whisper 100 (Tetra) into each carboy, in addition to a U-shaped plastic sampling hose, both of which remained in place for the entire experiment. Like the carboys, each of those were cleaned by overnight 1% bleach bath and extensively rinsed with deionized water before use.

To evaluate the decay of eNA over time, 2-liter water samples were collected from each carboy at nine timepoints: 0, 4, 8, 12, 24, 48, 96, 140, and 188 h (Fig. 1a). Sampling intervals were designed to densely capture rapid early decay (0–12 h) while still including later timepoints (48–188 h) to track long-lived eNA. This staggered approach minimizes the leverage of distant points in decay model fitting. At each timepoint, aeration was temporarily turned off to prevent aerosolization, and water was drawn via the plastic sampling hoses. We used sequential filtration to capture eNA across different particle sizes. Each sample was filtered through a tandem system of three filter housings (Smith-Root, non-self-preserving) equipped with 5 μm, 1.0 μm, and 0.45 μm mixed cellulose ester (MCE) 47 mm filters (Advantec), each backed by polyester drain disks (Sterlitech). Filters were connected downstream to an eDNA Citizen Science Sampler (Smith-Root) for vacuum filtration, and the filtering process took between 8 and 15 min per sample. Immediately after filtration, filter housings were opened, and sterilized forceps were used to fold each filter twice, sample side in, before placing it into 5 mL Lo-Bind tubes (Eppendorf) containing 2 mL of DNA/RNA Shield buffer (Zymo). Samples were incubated at room temperature for 30 min for standardization between replicates, then frozen at −80 °C until DNA extraction within one month. Decay rate was calculated considering each carboy’s water sampling time and each sample’s actual filtration time.

To prevent cross-contamination, all forceps, filter holders, adapters, and tubing were submerged in 5% bleach baths, thoroughly rinsed with deionized (DI) water, and dried before reuse. Two independent sets of filtration equipment were available, allowing for alternating use between timepoints. Additionally, at the end of each timepoint, we filtered 2 L of the DI water used for post-bleach rinsing through a 0.45 μm filter as a contamination control to verify the effectiveness of our decontamination procedures.

### DNA and RNA extraction

Nucleotides were extracted from samples using a two-step process. First, frozen filters with buffer were thawed and incubated with agitation at 37 °C for ~ 15 min to dissolve any precipitation. The samples were then thoroughly vortexed, and the buffer (2 mL) was transferred to an Amicon Ultra-15 30 kDa centrifugal filter unit (Millipore-Sigma) and centrifuged at 4,000 rpm for 30 min, concentrating the buffer to ~ 400 µL. Both eDNA and eRNA were extracted simultaneously using the Quick DNA/RNA Miniprep Kit (Zymo Research), following the manufacturer’s protocol with a 30-minute proteinase K incubation before adding the binding buffer.

This extraction process results in two sequential extracts (Fig. S2a). The first primarily contains the DNA (hereafter “eDNA”), whereas the second is expected to contain RNA; however, empirical results showed that it also contained a substantial amount of DNA, hereafter referred to as “carryover eDNA”. One-third of this second extract —containing both eRNA and carryover eDNA— was retained for carryover eDNA analysis (see supplement, Figs. S2, S3), while the remaining two-thirds were treated with ezDNAse (ThermoFisher Scientific) at 37 °C for 30 min to remove the carryover DNA in preparation for cDNA synthesis.

The DNAse-treated portion of the extract was subjected to a SuperScript IV (ThermoFisher Scientific) first-strand cDNA synthesis reaction using random hexamers in the denaturation step following the manufacturer’s protocol, to convert RNA into cDNA. This product is hereafter referred to as “eRNA.” Each sample was also subjected to a second control reaction, identical in components and cycling conditions, but without the reverse transcriptase enzyme. This reaction product is hereafter referred to as the “No-RT,” and it was used to assess post-DNAse treatment carryover eDNA, ensuring that quantifications from the eRNA extract were from eRNA-derived cDNA rather than residual carryover eDNA. For details on carryover eDNA analysis, see the supplementary material.

### Target eNA quantification

Target eNA were quantified using three assays, all of which target mitochondrial loci (Table 1, Table S1). The first was a 79 bp fragment of *Cytochrome b* (Cytb), a protein-coding gene, from which we quantified three components: eDNA, emRNA, and the No-RT control. The second was a 146 bp fragment of the *16 S* ribosomal RNA gene, used to quantify eDNA, erRNA, and No-RT control. The third was a 390 bp fragment of the *D-loop* control region, from which only eDNA was analyzed as it is a non-coding region.

In addition to these three markers quantified directly by their respective primers and assays, a fourth mitochondrial DNA (mtDNA) component—referred to as the “16S-Dloop bridge sequence”, hereafter “Bridge” for short—was quantified within the duplex ddPCR reaction targeting both *16 S* and *D-loop*. This Bridge signal arises from intact (2746 + bps) mtDNA molecules that physically link the two markers (Fig. 1b, see details below and in the supplement). Altogether, this yielded six distinct eNA components to be analyzed: Cytb eDNA, Cytb emRNA, 16 S eDNA, 16 S erRNA, Dloop eDNA, and Bridge eDNA (Table 1).

All markers were quantified using digital droplet PCR (ddPCR). Cytb eDNA, Cytb emRNA, Cytb No-RT, 16 S erRNA and 16 S No-RT were quantified using monoplex reactions. Each 22 µL reaction contained 11 µL of ddPCR Supermix for Probes (Bio-Rad Inc., Hercules, CA), 250 nM of probe, 900 nM of each primer (IDT), and 2 µL of template extract. The thermocycling conditions were: 4 °C for 10 min; 95 °C for 10 min; 45 cycles of 94 °C for 30 s and 60 °C for 60 s (annealing/extension); followed by 98 °C for 10 min and a final hold at 4 °C.

For *16 S* and *D-loop* eDNA, we used a duplex ddPCR reaction. Each 22 µL reaction included 11 µL of Supermix, 900 nM of *D-loop* primers, 600 nM of *16 S* primers, 250 nM of each probe, and 2 µL of extract. Given the longer amplicon sizes, we adjusted cycling conditions: 4 °C for 10 min; 95 °C for 10 min; 45 cycles of 94 °C for 30 s, 56 °C for 30 s (annealing), and 72 °C for 120 s (extension); followed by 98 °C for 10 min and a 4 °C hold. This duplex design allowed us to simultaneously quantify 16 S, D-loop, and the mtDNA bridge between them (Fig. 1b).

As mentioned above, this duplex reaction also allowed us to quantify a fourth marker, Bridge, given by ddPCR droplet sorting and linkage between markers (Fig. S1). Droplet PCR partitions the reaction into ~ 20,000 droplets, enabling absolute quantification of target molecules using Poisson statistics. If targets are unlinked, they sort into droplets independently, and double-positive droplets arise randomly. However, due to the circular nature of mtDNA, *16 S* and *D-loop* are physically linked in intact molecules. The shortest span between them is 2746 bp, though linkage could span the full ~ 16,388 bp mitochondrial genome [66,67]. Thus, in a hypothetical situation where all mtDNA molecules are intact in the sample, all droplets containing 16 S would automatically also contain Dloop, and vice versa, making all droplets positive for both markers.

However, because eDNA in environmental samples is partially fragmented, droplets in the duplex assay include a mixture of single-positive and double-positive events. Double-positive droplets can arise from two mechanisms: (1) random co-localization of independently degraded DNA fragments containing 16 S and D-loop sequences, or (2) relatively intact DNA molecules physically bridging the two markers (i.e., ≥ 2746 bp). Using the counts of single-positive droplets for each marker, one can calculate the expected number of double-positive droplets that would result from mechanism (1) alone. This expected value is then subtracted from the observed number of double-positive droplets, and the remainder is attributed to mechanism (2), providing an estimate of the number of relatively intact, bridged molecules. See supplements for full derivation and schematics.

For all ddPCR assays, droplets were generated using the AutoDG Droplet Generator (Bio-Rad Inc.), thermocycling was performed on C1000 Touch Thermal Cyclers (Bio-Rad Inc.), and droplet fluorescence was read using a QX200 Droplet Reader (Bio-Rad Inc.). Each plate included one positive control containing DNA extracted from target species and three no-template controls (NTCs), used to define positive thresholds and screen for contamination.

Finally, to confirm the absence of non-target marine mammals in the decay experiment, we opportunistically performed metabarcoding on eDNA samples filtered at the first timepoint of each carboy. We detected no other cetacean species in the decay water (Tables S2, S3). Details of metabarcoding methods and results are provided in the Supplement.

### Statistical analysis and decay rate Estimation

We built a Bayesian hierarchical model to jointly estimate the eNA concentrations and the decay rates for each component and marker combination *i* (Table 1) from the observed ddPCR droplet counts (W). To estimate the eNA concentrations from ddPCR droplet reader observations, we followed the same approach used in Guri et al.^68^ where the number of positive droplets from technical replicates (*r*) of the same sample collected at time (*t*) are modeled as a Binomial distribution:

1 $$\:\begin{array}{c}{W}_{irt}\:\sim\:Binomial\:\left({U}_{irt},\:{p}_{it}\right)\end{array}$$

2 $$\:\begin{array}{c}cloglog\left({p}_{it}\right)=ln\left({\omega\:}_{it}\right)\end{array}$$

where $\:\:{p}_{it}$is the probability that an individual droplet positively amplifying the target locus *i* at sampled time *t* from the total number of droplets generated *U*_*irt*_ and *ω*_*it*_ is the respective eNA molecules (in copies/µL reaction volume).

For inferring the eNA concentration in the mesocosm (*C*_*i*_; units copies/L) we normalize for the volume filtered, eluted, and diluted as follows:

3 $$\:\begin{array}{c}ln\left({\omega\:}_{it}\right)={ln}\left({C}_{it}\right)-\:{ln}\left(rvol\right)+\:{ln}\left(tvol\right)+ln\left({d}_{it}\right)-\text{ln}\left(evol\right)-ln\left({filt}_{it}\right)-\:{ln}\left(dvol\right)\end{array}$$

where *rvol* is the total ddPCR reaction volume (22 µL), $\:tvol\:$is the template volume added to the reaction (2 µL), $\:{d}_{i}$ is a sample-specific template dilution (caused by reverse transcription reaction), *evol *is the extraction eluted volume (50 µL), $\:{filt}_{i}$ is the sample-specific filtered volume, and *dvol* is the ddPCR droplet volume (~ 0.85 nL), as specific by Bio-Rad and used by Guri et al. (2024).

To calculate decay for each of the components (c), we tested six different decay models and ultimately used the Biphasic Exponential Decay model which yielded the highest likelihood (see Supplements for model testing). The biphasic exponential decay model assumes two exponential decay phases with a transition time specific for each component and marker (t_ix_):

4 $$\:\begin{array}{c}{\text{C}}_{cmt}=\left\{\begin{array}{c}{\text{C}}_{\text{i},\text{t}=0}{\times\:e}^{-{{\uplambda\:}1}_{\text{i}}\times\:\text{t}}\:,\:\:\:\:\:\:\:\:\:t<\:{\text{t}}_{\text{i}\text{x}},\\\:{\text{C}}_{\text{i},\text{t}={\text{t}}_{ix}}{\times\:e}^{-{{\uplambda\:}2}_{\text{i}}\times\:\left(t-{t}_{ix}\right)}\:,\:t\:\ge\:\:{\text{t}}_{\text{i}\text{x}}.\end{array}\right.\end{array}$$

where $\:{\text{C}}_{it}$ is the eNA concentration of each component (*c*) and marker (*m*) at time (*t*); $\:{\text{C}}_{\text{i},\text{t}=0}$ is the respective initial concentration at t = 0; $\:{{\uplambda\:}1}_{\text{i}}$ and $\:{{\uplambda\:}2}_{\text{i}}$ are the decay rate constants for the first and second phases, respectively; $\:{\text{t}}_{\text{i}\text{x}}$ is the time where decay rate changes; and $\:{\text{C}}_{\text{i},\text{t}={\text{t}}_{ix}}$ is the concentration at $\:{\text{t}}_{\text{i}\text{x}}$ determined as $\:{\text{C}}_{cm,t={t}_{ix}}={\text{C}}_{\text{i},\text{t}=0}\times\:{e}^{-{{\uplambda\:}1}_{\text{i}}\times\:{\text{t}}_{ix}}$.

The concentrations $\:{\text{C}}_{cmt}$ are jointly modeled from two technical replicates (r), and the decay rates ($\:{{\uplambda\:}1}_{\text{i}}$ and $\:{{\uplambda\:}2}_{\text{i}}$) and $\:{\text{t}}_{\text{x}}$ for each component and marker combination considering all three biological replicates. The model was implemented in the Stan language with the R package Rstan^69^ running four independent Markov chain Monte Carlo (MCMC) chains with 5000 warmup and 10,000 sampling iterations. Model’s effective sample size was above 500, and model’s $\:\widehat{R}$ convergence parameter was < 1.005 for all estimated parameters.

Finally, we computed two molecular ratios as putative time-sensitive indicators (“molecular clocks”): (i) the proportion of long (Bridge) vs. short (Cytb) mitochondrial fragments, and (ii) the proportion of ribosomal eRNA relative to total nucleic acids (16 S marker). Using posterior samples from our hierarchical biphasic decay model, we predicted concentrations of each molecular component across a 0–24 h time window. From each posterior draw, we computed the corresponding ratio across time, then summarized the median and 95% credible interval for each time point. As a heuristic summary of trend direction and magnitude, we fit a linear regression to the posterior median trajectory of each ratio.

## Electronic supplementary material

Below is the link to the electronic supplementary material.

Supplementary Material 1

Supplementary Material 2

Supplementary Material 3

## Data Availability

Raw dataset with ddPCR quantifications (csv) and corresponding metadata are available as supplement files.

## Declarations

### Supporting information

Additional description of ddPCR methods, additional visualizations of the raw data, and analysis of carryover eDNA is provided in a pdf as Supplementary Information.
